# Retinal thinning in progressive supranuclear palsy: differences with healthy controls and correlation with clinical variables

**DOI:** 10.1007/s10072-022-06061-4

**Published:** 2022-04-11

**Authors:** Marina Picillo, Giulio Salerno, Maria Francesca Tepedino, Filomena Abate, Sofia Cuoco, Marco Gioia, Alessia Coppola, Roberto Erro, Maria Teresa Pellecchia, Nicola Rosa, Paolo Barone, Maddalena De Bernardo

**Affiliations:** 1 Center for Neurodegenerative Diseases (CEMAND), Department of Medicine, Surgery and Dentistry “Scuola Medica Salernitana”, University of Salerno, Via Allende, 84081 Baronissi, Salerno Italy; 2 Eye Unit, Department of Medicine, Surgery and Dentistry “Scuola Medica Salernitana”, University of Salerno, Baronissi, Italy

**Keywords:** Progressive supranuclear palsy, Retina, Progression, Diagnosis, Optical coherence tomography

## Abstract

**Background:**

Available evidence reports conflicting data on retinal thickness in progressive supranuclear palsy (PSP). In studies including healthy controls, PSP showed either the thinning of the retinal nerve fiber layer, macular ganglion cell, inner nuclear, or outer retina layer.

**Objectives:**

The goals of the present study were to describe retinal layer thickness in a large cohort of PSP compared to healthy controls and in PSP phenotypes using spectral-domain optical coherence tomography (SD-OCT). The additional objective was to verify the relationship between retinal layers thickness and clinical variables in PSP.

**Methods:**

Using a cross-sectional design, we examined retinal structure in 27 PSP patients and 27 controls using standard SD-OCT. Motor and cognitive impairment in PSP was rated with the PSP rating scale and the Montreal Cognitive Assessment battery (MoCA), respectively. Eyes with poor image quality or confounding diseases were excluded. SD-OCT measures of PSP and controls were compared with parametric testing, and correlations between retinal layer thicknesses and disease severity were evaluated.

**Results:**

PSP showed significant thinning of the inner retinal layer (IRL), ganglion cell layer (GCL), inner plexiform layer (IPL), and the outer plexiform layer (OPL) compared to healthy controls. PSP phenotypes showed similar retinal layer thicknesses. Retinal layer thickness correlated with MoCA visuospatial subscore (*p* < 0.001).

**Conclusions:**

We demonstrated PSP patients disclosed thinner IRL, GCL, IPL, and OPL compared to healthy controls. Furthermore, we found a significant correlation between visuospatial abilities and retinal layers suggesting the existence of a mutual relationship between posterior cognitive function and retinal structure.

## Introduction

PSP is a rare, neurodegenerative tauopathy with relentless progression characterized by postural instability, oculomotor disturbances, akinesia, and cognitive deficits [1]. Based on the diverse combination of the core clinical features, different phenotypes of the disease have been described with the most common being Richardson’s syndrome (PSP-RS) [1].

Few neuroimaging markers have been reported to support the clinical diagnosis of PSP in comparison with healthy controls and other parkinsonian syndromes [2]. Complementary, no clinical or instrumental assessment showed sufficient reliability in discriminating between PSP-RS and the other variant syndromes of PSP (vPSP) [3–5].

Despite intense efforts from the research community, reliable in vivo biomarkers are lacking [1, 6]. Hence, a definite diagnosis and phenotypization of PSP still relies on pathological examination showing a high density of neurofibrillary tangles and neuropil threads in the basal ganglia and brainstem with a characteristic distribution associated with tau-positive astrocytes.

SD-OCT is a noninvasive, inexpensive technique providing high-resolution retinal imaging with elevated reproducibility. Given tau aggregates have been detected in retina layers in both mice expressing mutant tau and patients affected by PSP, in vivo imaging of the retina may represent a promising biomarker of PSP [7–9]. Yet, available evidence reports conflicting data on retinal thickness in PSP patients. In studies including healthy controls, PSP showed either thinning of retinal nerve fiber layer (RNFL), macular ganglion cell, inner nuclear, or outer retina layer [10–13]. However, several limitations including a limited number of patients examined and a lack of disease-specific clinical assessments hamper drawing any conclusion from previous studies on the importance of retinal thickness as an in vivo biomarker in PSP [10–13].

The goals of the present study were to evaluate retinal layer thickness in a large cohort of PSP compared to a healthy control group and in PSP phenotypes using SD-OCT. The additional objective was to verify the relationship between retinal layer thickness and clinical variables in PSP.

## Patients and methods

### Participants

Thirty-two patients diagnosed with PSP according to the Movement Disorder Society (MDS) criteria were invited to take part in the study and examined between June 2018 and December 2019 [1]. Detailed information on enrollment and application of the PSP diagnostic criteria to determine disease phenotype is available elsewhere [2–5]. Each patient contributed with one eye. Five patients were excluded for either incomplete data or confounding eye diseases. Thus, 27 PSP patients (27 eyes) were considered in the present analysis.

The severity of the disease was evaluated with the PSP rating scale (PSP-rs) total score and related subscores (mental, bulbar, ocular, limb, and gait), while cognitive abilities were rated with the MoCA and related subscores (visuospatial, executive, language, orientation, attention, and memory) [14, 15].

A group of 27 eyes from 27 sex-matched healthy controls (HC) with similar age and axial length, without diabetes, or other confounding eye diseases were enrolled (PSP:HC = 1:1). Healthy controls did not undergo formal neurological examination but had no history of any neurodegenerative disease.

### Imaging protocol and image analysis

All participants underwent SD-OCT imaging with the Heidelberg SPECTRALIS (Heidelberg Engineering, Heidelberg, Germany) with a standard macular volume scan protocol and obtained the segmentation of the retinal layers using the instrument’s automatic algorithm. Poor quality images with a signal-to-noise score lower than 25 dB were excluded.

Using this system, 11 optical interfaces were obtained for the study of the 10 retinal layers [10]; using the standard Early Treatment Diabetic Retinopathy Study (ETDRS) grid, the thickness of the retinal layers at the circle centered on the fovea (1 mm in diameter), and the average of the 5 foveal and parafoveal zones (3 mm in diameter) were studied.

**Fig. 1:**
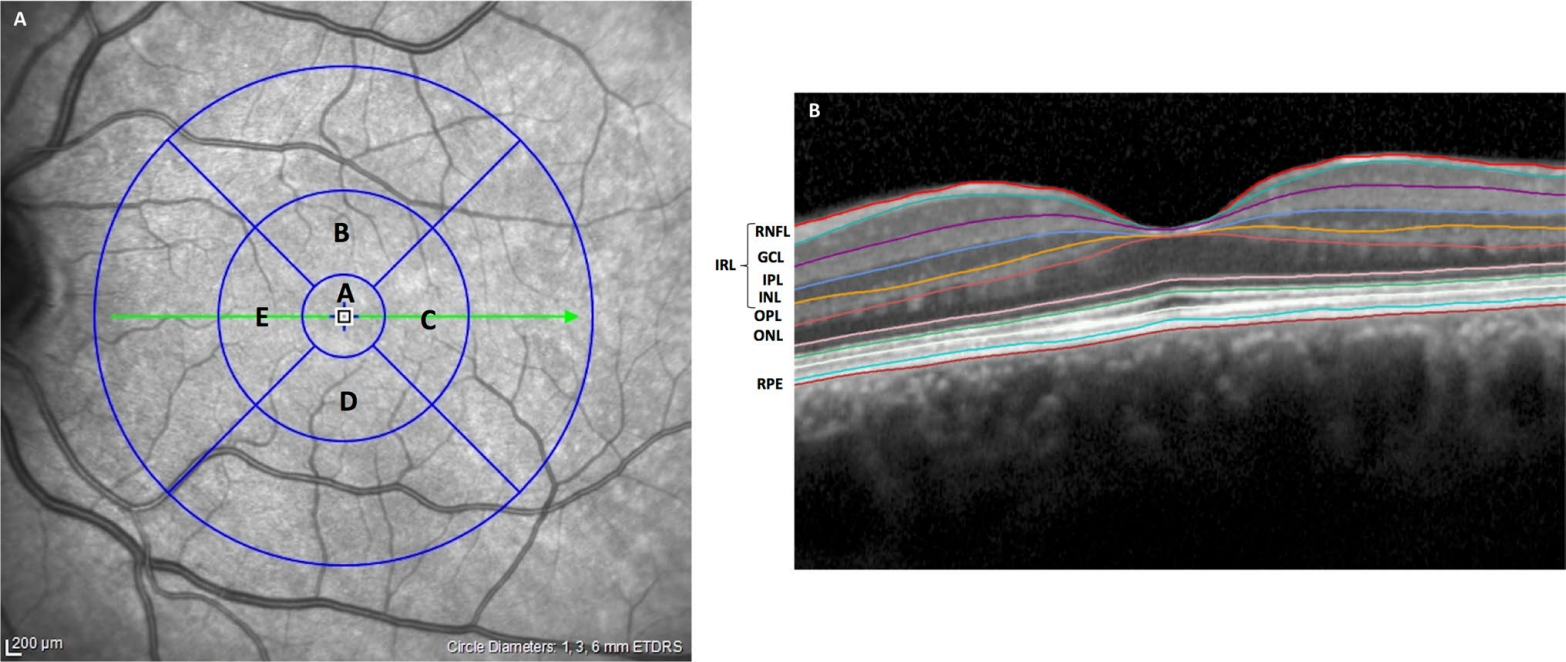
**A **ETDRS grid. **A** central circle at the fovea (diameter 1 mm). A + B + C + D + E = 5 central regions (3 mm diameter). Abbreviation: EDTRS: Early Treatment Diabetic Retinopathy Study. **B** Segmentation of the retinal layers using the instrument’s automatic algorithm. Abbreviations: GCL, ganglion cell layer; INL, inner nuclear layer; IPL, inner plexiform layer; IRL, inner retinal layer; ONL, outer nuclear layer; OPL, outer plexiform layer; RNFL, retinal nerve fiber layer; RPE, retinal pigment epithelium

For the fovea (1 mm diameter), the values of the total thickness (total retina), photoreceptor layer, retinal pigment epithelium (RPE), outer nuclear layer (ONL), OPL, ONL/OPL ratio, and the IRL thickness were collected [16, 17]. This last value includes the sum of RNFL, the GCL, the IPL, and inner nuclear (INL) layers that are closely set together at the foveal center. For the parafoveal zone (3 mm diameter), however, the thickness value for these layers was also evaluated individually. Figure 1A shows the EDTRS grid and the 5 foveal and parafoveal zones; Fig. 1B outlines the segmentation of the retinal layers.

The eyes of PSP patients and controls were evaluated with an IOL Master (5.4.4.0006; Carl Zeiss Meditec AG) to measure the axial length and to match the results. The mean of at least three measurements with the highest signal-to-noise ratio at least above 2 was considered.

### Statistical analysis

After checking for normal distribution of data with the Kolmogorov–Smirnov test, parametric testing was used to compare the thickness measures of retinal layers between PSP and healthy controls. Then, the layers showing significant differences between groups were analyzed with the area under the receiver operating characteristics (ROC) to quantify their ability to discriminate between patients and controls.

The relationship between retinal layers and cognitive (MoCA total score and subscores) and motor (PSP-rs total score and subscores) measures was evaluated with Pearson’s correlation. Multiple comparisons were corrected with the Bonferroni test (*p* < 0.001).

Statistical analyses were performed with SPSS v.23 and a two-sided *p* < 0.05 was considered statistically significant.

### Standard protocol approvals, registrations, and patient consent

This study was approved by the local ethics committee, and all participants were included upon signature of the written informed consent form.

## Results

Demographic characteristics and axial length of enrolled PSP and HC are detailed in Table 1. Enrolled PSP had a disease duration of 2.41 ± 1.08 years. Seventeen patients (62.9%) presented PSP-RS, and 10 presented the other variant syndromes of PSP (vPSP) including 6 PSP with predominant parkinsonism and 4 PSP with progressive gait freezing.

**Table 1:** Demographic features and axial length of PSP and HC

	PSP (27)	HC (27)	*p*
Age, mean ± SD (min, max)	69.9 ± 7.2 (53, 81)	69.2 ± 11.3 (37, 86)	0.799
Sex, *n* (%) men	16 (59.3)/11 (40.7)	16 (59.3)/11 (40.7)	1.000
Axial length, mean ± SD (min, max)	23.19 ± 0.85 (21.42, 25.34)	23.18 ± 0.85 (21.5, 25.43)	0.944
Right eye, *n* (%)	14 (51.9)	14 (51.9)	1.000

Abbreviations: *HC*, healthy controls; *M*, men; *PSP*, progressive supranuclear palsy; *SD*, standard deviation

**Fig. 2:**
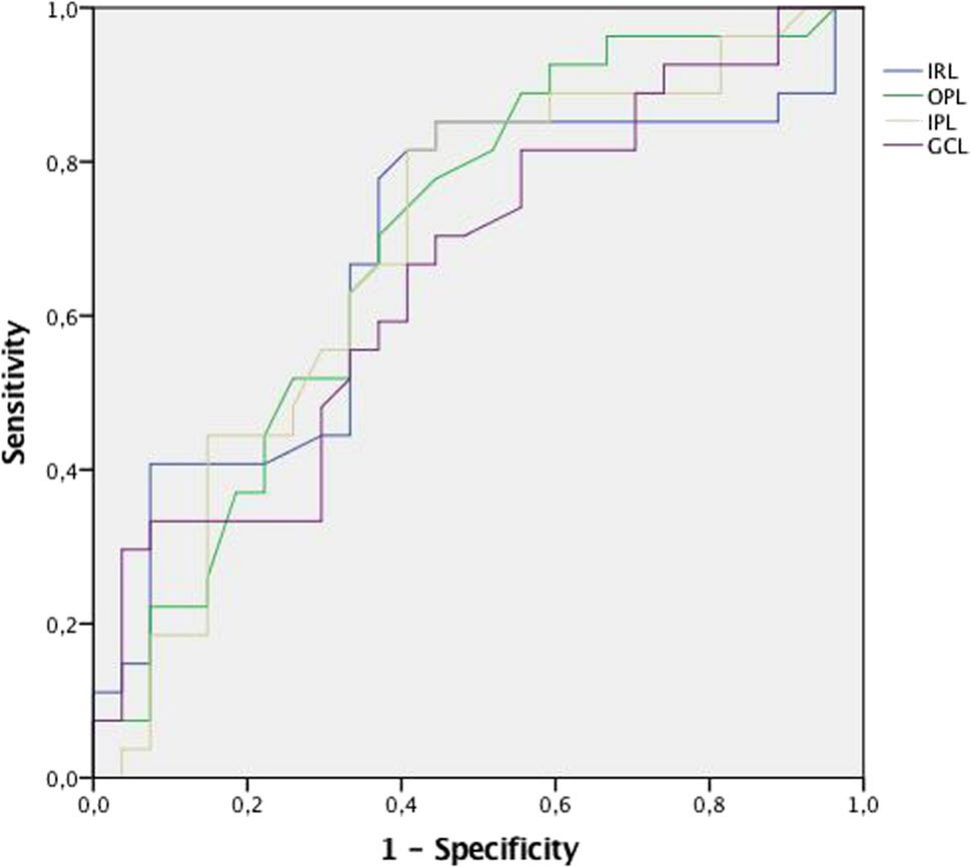
Results from the ROC analysis for IRL, OPL, IPL, and GCL from the central 5 regions. *Y* axis: sensitivity. *X* axis: specificity. IRL, inner retinal layer; OPL, outer plexiform layer; IPL, inner plexiform layer; GCL, ganglion cell layer

Table 2 shows the central circle at the fovea (diameter 1 mm) and the average of the central 5 regions (diameter 3 mm) for PSP and HC. When considering the central 5 regions, PSP patients showed significant thinning of the IRL, OPL, IPL, and GCL compared to HC (Table 2). The ROC analysis for discriminating patients from controls found that the area under the curve (AUC) was 0.684 (95% confidence interval 0.536–0.831) for IRL, 0.698 (95% confidence interval 0.556–0.839) for OPL, 0.689 (95% confidence interval 0.689 to 0.834) for IPL and 0.655 (95% confidence interval 0.509–0.801) for GCL (Fig. 2). PSP-RS showed similar retinal thicknesses as vPSP (Table 3). PSP-RS and vPSP did not present any difference in terms of age, sex distribution, and axial length (data not shown).

**Table 2:** Comparisons of retinal layer thicknesses (μm) between PSP and HC

	PSP (27)	HC (27)	*p*
Fovea (diameter 1 mm)			
Total retina	268.3 ± 21.4	271.4 ± 19.8	0.582
Photoreceptor	87.6 ± 5.54	85 ± 5	0.222
IRL	181.6 ± 22.6	186.3 ± 19.4	0.413
RPE	15.4 ± 1.7	14.8 ± 1.3	0.144
ONL	87.8 ± 10.6	90.4 ± 16	0.558
OPL	24.3 ± 5.3	25.8 ± 6.3	0.346
ONL/OPL	3.8 ± 1.1	3.7 ± 1.3	0.750
5 central regions (3 mm)			
Total retina	314.9 ± 14.8	322 ± 23.4	0.127
Photoreceptors	82.5 ± 3.8	81.6 ± 3.4	0.412
IRL	232.4 ± 14.9	247.1 ± 27.7	**0.019**
RPE	15 ± 1.7	14.5 ± 1.2	0.211
ONL	72.9 ± 7	75 ± 11.7	0.428
OPL	29.8 ± 3	31.8 ± 3.1	**0.024**
INL	37.4 ± 3.4	38.6 ± 3.7	0.235
IPL	34.1 ± 4.3	36.2 ± 3.3	**0.046**
GCL	38.7 ± 5.9	41.9 ± 4.9	**0.037**
RNFL	19.6 ± 2.4	20.2 ± 1.7	0.344
ONL/OPL	2.4 ± 0.3	2.4 ± 0.4	0.441

Data are shown in mean ± standard deviation. Abbreviations: *GCL*, ganglion cell layer; *HC*, healthy controls; *INL*, inner nuclear layer; *IPL*, inner plexiform layer; *IRL*, inner retinal layer; *ONL*, outer nuclear layer; *OPL*, outer plexiform layer; *PSP*, progressive supranuclear palsy; *RNFL*, retinal nerve fiber layer; *RPE*, retinal pigment epithelium. Significant values are reported in bold

**Table 3:** Comparisons of retinal layer thicknesses (μm) between PSP-RS and vPSP

	PSP-RS (17)	vPSP (10)	*p*
Fovea (diameter 1 mm)			
Total retina	267.7 ± 22.4	269.4 ± 20.5	0.852
Photoreceptor	87.7 ± 4.7	85.1 ± 6.5	0.235
IRL	180.1 ± 24.1	184.2 ± 20.9	0.660
RPE	15.5 ± 1.8	15.2 ± 1.6	0.585
ONL	85.7 ± 11.2	91.4 ± 8.8	0.189
OPL	25.1 ± 5.2	23 ± 5.4	0.314
ONL/OPL	3.5 ± 0.89	4.2 ± 1.4	0.137
5 central regions (3 mm)			
Total retina	312.2 ± 15.4	318.7 ± 13.7	0.325
Photoreceptors	83.2 ± 3.4	81.3 ± 4.4	0.227
IRL	229.6 ± 15.3	237.2 ± 13.5	0.209
RPE	15.1 ± 1.8	14.8 ± 1.6	0.647
ONL	72.6 ± 8.1	73.4 ± 5.1	0.787
OPL	29.7 ± 3.4	30 ± 2.4	0.787
INL	37.7 ± 3.6	36.9 ± 3.2	0.574
IPL	32.9 ± 4.4	36 ± 3.5	0.075
GCL	37.5 ± 6.5	40.7 ± 4.2	0.186
RNFL	19.2 ± 2.2	20.4 ± 2.7	0.202
ONL/OPL	2.4 ± 0.4	2.4 ± 0.2	0.871

Data are shown in mean ± standard deviation. Abbreviations: *GCL*, ganglion cell layer; *INL*, inner nuclear layer; *IPL*, inner plexiform layer; *IRL*, inner retinal layer; *ONL*, outer nuclear layer; *OPL*, outer plexiform layer; *PSP-RS*, progressive supranuclear palsy with Richardson’s syndrome; *RNFL*, retinal nerve fiber layer; *RPE*, retinal pigment epithelium; *vPSP*, the other variant syndrome of PSP

In PSP, MoCA visuospatial subscore was positively correlated with IRL and total retinal thickness of the fovea (*r* = 0.565, *p* = 0.004 and *r* = 0.563, *p* = 0.004, respectively) and with IRL (*r* = 0.660, *p* < 0.001), OPL (*r* = 0.565, *p* = 0.004), INL (*r* = 0.702, *p* < 0.001), IPL (*r* = 0.556, *p* = 0.005), GCL (*r* = 0.496, *p* = 0.014), total retina thickness (*r* = 0.636, *p* < 0.001) of the 5 central regions. As for motor variables, photoreceptor of the fovea was negatively correlated with PSP-rs bulbar subscore (*r* =  − 0.413, *p* = 0.036) and IRL and total retinal thickness of the 5 central regions with PSP-rs limb subscore (*r* =  − 0.388, *p* = 0.045 and *r* =  − 0.403, *p* = 0.037, respectively). Also, the photoreceptor of the 5 central regions was negatively correlated with PSP-rs bulbar subscore (*r* =  − 0.538, *p* = 0.004) and IPL and GCL of the 5 central regions with PSP-rs gait subscore (*r* =  − 0.438, *p* = 0.022 and *r* =  − 0.493, *p* = 0.009, respectively). No other significant correlations were detected. The correlation between MoCA visuospatial subscore and IRL, INL, and total retina of the 5 central regions remained significant after correction for multiple comparisons (*p* < 0.001).

## Discussion

Herein, we showed PSP is associated with the thinning of IRL, OPL, IPL, and GCL compared to healthy controls in all the five central regions, except in the foveal central circle. Such difference was present only when considering the average of the 5 central regions and not when considering the central circle at the fovea. The reduced representation of retinal layers in the foveal central circle, mainly concerning the IRL, may account for such findings.

Few studies investigated retinal layers’ thickness by SD-OCT in PSP patients showing a thinning of INL and ONL compared to other forms of parkinsonism [16, 17]. In contrast with our findings, Schneider et al. found thicker OPL in PSP patients [17]. Such discrepancy between our findings and previous data may be explained in part by differences in the segmentation method of the retinal layers. As such, while Albrecht et al. carried out a manual segmentation of the parafoveal retinal layers and Schneider et al. used a semiautomatic algorithm, in the present study, we adopted an automatic segmentation [16, 17]. More recently, Woo et al. found thinner peripapillary RNFL in 21 PSP compared to 22 HC [18]. As opposite to this study, ours included a 1:1 match between PSP and HC eyes also taking into account axial length [18].

Both in vivo and postmortem studies suggest a decreased number of ganglion cells and an RNFL thinning, and in general of the inner retina, in Alzheimer’s disease [19, 20]. On the other hand, scant data is available on retinal thickness in non-Alzheimer’s dementia. Recent evidence would support a selective thinning of the outer retina in patients affected by frontotemporal degeneration (FTD) clinical syndromes [10]. In detail, Kim et al. reported the outer retina thinning driven by the ONL thinning in 27 FTD patients, including 12 PSP, compared to healthy controls [10]. Contradicting previous findings, our study does not support this simplistic differential involvement of retinal layers according to the dementia type, i.e., Alzheimer’s disease associated with inner retina thinning and FTD with outer retina thinning [10]. Instead, our data would support a patchy involvement of retinal layers in PSP, involving both the inner (i.e., IRL) and outer (i.e., OPL) retina, more evident when considering the average of the 5 central regions than the central foveal circle alone.

Several reasons may account for such discrepancies with previous data [10]. First, our study is focused on PSP only and involves a larger number of patients (27 versus 12). Second, we compared retinal layer thicknesses between PSP and a group of healthy controls with similar age and sex distribution. But more importantly, our PSP and healthy controls showed comparable axial length, ensuring the reliability of retinal layers comparisons between groups. As a matter of fact, evidence demonstrates that axial length may influence the measurement of retinal and choroidal thickness [21, 22], and none of the previously published studies on this topic compared patients and controls according to these parameters. Finally, all our patients and healthy controls were Caucasian. On the contrary, Kim et al. included a group of healthy controls not matched for age, ethnicity, and axial length with FTD [10].

Our results were also supported by the ROC analysis, which demonstrated a fair diagnostic accuracy for both IRL, GCL, IPL, and OPL in discriminating between PSP and healthy controls (Fig. 2).

Using the immunoreactivity technique, previous studies showed tau protein accumulation in older individuals’ human retinal layers, such as RNFL, INL, IPL, and OPL [7, 8]. Considering that PSP is a neurodegenerative tauopathy and the RNFL, INL, and IPL layers are part of the IRL, we speculate that accumulation of tau protein in such retinal layers may cause the IRL, GCL, IPL, and OPL degeneration in PSP patients, with possible evolution in retinal thinning [6].

Similar to recent data [18], we failed to detect differences in retinal thickness between PSP phenotypes (PSP-RS vs vPSP) in any layer. Indeed, while IRL, GCL, IPL, and OPL proved able to differentiate PSP from healthy controls, none of the retinal thicknesses presented significant differences among MDS PSP subtypes (Table 3). The MDS PSP phenotypes were recently conceived based on an extensive review of the literature as well as the revision of the largest autopsy-confirmed case series reported so far [1]. In spite of being considered a window on brain changes, retinal thickness adds to the list of in vivo clinical and neuroimaging assessments not supporting the differentiation of the PSP phenotypes [2–5]. In such a scenario, there is a dearth of in vivo biomarkers supporting specific phenotypic attribution.

As for the correlation between retinal thickness and disease severity in PSP, we demonstrated a significant relationship between MoCA visuospatial subscore and IRL, INL, and the total retinal layer of the 5 central regions, which remained statistically significant after correcting for multiple comparisons. Retinal thickness has been associated with cognitive performances in tests requiring visual processing in other neurological conditions including multiple sclerosis [23]. Such association may have two possible explanations. First, greater retinal thinning may result in subclinical vision difficulties, which in turn affect the performance of cognitive testing requiring visual processing. Alternatively or complementary, greater retinal thinning may represent a marker of a more severe form of disease manifesting with poorer cognitive functions [23–25]. Since we missed to assess visual acuity and visual field, as well as perform neuroimaging studies in the present study, we cannot draw firm conclusions, and further studies are needed to clarify this aspect.

As for the relationship with motor scores, the correlation between PSP-rs bulbar, limb, and gait subscores with retinal layers lost significance after correction for multiple comparisons. Indeed, motor impairment in PSP is associated with a wide range of determinants possibly explaining the weaker relationship with retinal thickness.

In comparison with previous data [10–13], the strengths of the present study include a larger sample size of the PSP cohort, the comparison of PSP with a control group with similar age, sex and axial length, and the use of disease-specific clinical assessments (i.e., the PSP rating scale and the MoCA).

In conclusion, we demonstrated PSP patients disclose thinner IRL, GCL, IPL, and OPL compared to sex-matched healthy controls with similar age and axial length. Furthermore, we found a significant correlation between visuospatial abilities and retinal layers suggesting the existence of a mutual relationship between posterior cognitive function and retinal structure.

## Abbreviations

MDS: Movement Disorder Society
MoCA: Montreal Cognitive Assessment battery
OCT: Optical coherence tomography
PSP: Progressive supranuclear palsy
PSP-rs: Progressive supranuclear palsy rating scale
PSP-RS: Progressive supranuclear palsy with Richardson’s syndrome
RNFL: Retinal nerve fiber layer
ROC: Receiver operating characteristics
vPSP: The other variant syndromes of progressive supranuclear palsy
